# Assessing Deep-Pelagic Shrimp Biomass to 3000 m in The Atlantic Ocean and Ramifications of Upscaled Global Biomass

**DOI:** 10.1038/s41598-019-42472-8

**Published:** 2019-04-11

**Authors:** Alexander L. Vereshchaka, Anastasia A. Lunina, Tracey Sutton

**Affiliations:** 1 Shirshov Institute of Oceanology, Russian Academy of Sciences, Nakhimov Pr. 36, Moscow, 117997 Russia; 2 Halmos College of Natural Sciences and Oceanography, Nova Southeastern University, Dania Beach, FL 33004 USA

## Abstract

We assess the biomass of deep-pelagic shrimps in the Atlantic Ocean using data collected between 40°N and 40°S. Forty-eight stations were sampled in discrete-depth fashion, including epi- (0–200 m), meso- (200–800/1000 m), upper bathy- (800/1000–1500 m), and lower bathypelagic (1500–3000 m) strata. We compared samples collected from the same area on the same night using obliquely towed trawls and large vertically towed nets and found that shrimp catches from the latter were significantly higher. This suggests that vertical nets are more efficient for biomass assessments, and we report these values here. We further compared day and night samples from the same site and found that biomass estimates differed only in the epi- and mesopelagic strata, while estimates from the bathypelagic strata and the total water column were independent of time of day. Maximal shrimp standing stocks occurred in the upper bathypelagic (52–54% of total biomass) and in the mesopelagic (42–43%). We assessed shrimp biomass in three major regions of the Atlantic between 40°N and 40°S, and the first-order extrapolation of these data suggests that the global low-latitude deep-pelagic shrimp biomass (1700 million tons) may lie within the range reported for mesopelagic fishes (estimations between 1000 and 15000 million tons). These data, along with previous fish-biomass estimates, call for the reassessment of the quantity and distribution of nektonic carbon in the deep ocean.

## Introduction

Pelagic shrimps are an important component of the deep-pelagic ecosystems. The dominant (numerical and biomass) components are the families Sergestidae, Benthesicymidae, Acanthephyridae, and Oplophoridae. As the deep-pelagic domain accounts for nearly 95% of the habitable volume of the World Ocean^1^, the biomass of its main components, including shrimps, merits accurate assessment. It has been long believed that pelagic shrimps, which are one trophic level higher than the mesozooplankton, have a biomass one order of magnitude lower than the mesozooplankton, in accordance with the traditional Eltonian biomass pyramid^2^. Further, this belief has been supported by various sources of shrimp biomass data, traditionally assessed using horizontally or obliquely towed, opening-closing trawls, such as the Isaacs-Kidd midwater trawl^3,4^, MOCNESS^5^, and BIONESS^6^. The use of these gears provides taxon-specific biomass values standardized by effort (volume filtered), and in the layers of maximal concentration, suggest standing stock biomasses in the range of 0.1–0.5 mg m^−3^ in the Atlantic^3,4^, Indian^7^, and Southeast Pacific Oceans^8^. Vertically hauled nets, such as the Bogorov-Rass net (1-m^2^ opening), have been widely used for mesozooplankton sampling^9–11^, but micronektonic shrimps were generally not quantified in the catches due to the belief that the net mouth area was too small, resulting in avoidance by shrimps. Surprisingly, later analyses of data from these types of nets provided higher, not lower, estimates of shrimp biomass; recent studies revealed biomass estimates at least an order of magnitude higher than was previously thought^12^. The authors explained this finding by the upward-directed escape behavior of pelagic shrimps, which would be more effective at avoiding horizontal and oblique gears but less so in the case of vertical gears^12^. The same study showed that shrimp biomass may be correlated with surface chlorophyll concentrations as measured remotely via satellites and that the shrimp biomass standing stock can be estimated for extensive oceanic areas.

In previous papers^12,13^, we divided deep-pelagic trawl samples into three major taxonomic groups, with pelagic decapods (i.e. shrimps) being one. In contrast to the mesozooplankton data, the shrimp biomass estimates were considered quasi-quantitative because of three potential issues: (1) sample size (usually few individuals per haul vs. hundreds of zooplankton individuals); (2) lack of precedence using vertical net hauls, not oblique trawls as before, to estimate shrimp biomass; and (3) pelagic shrimps are strong migrants and sampling time, day or night, may significantly affect abundance and biomass values in some layers and in the whole water column. In order to address the first problem, here we have added 103 new deep-sea samples, doubling the size of the deep-pelagic decapod shrimp database. Second, we have conducted an exhaustive comparison of nearly synchronous hauls by vertical and oblique trawls at ten stations. Finally, we have compared 52 day and night net samples taken at 13 sites to see which strata are affected by vertical migrations and which are not. As the new dataset allows a more robust assessment of the deep-pelagic shrimp biomass standing stock, we compare resulting values with those obtained for mesopelagic fishes^14–16^ to provide a new perspective on the abundance of deep-pelagic nekton and the relative contribution of pelagic decapods.

## Material and Methods

### Net samples

**Figure 1:**
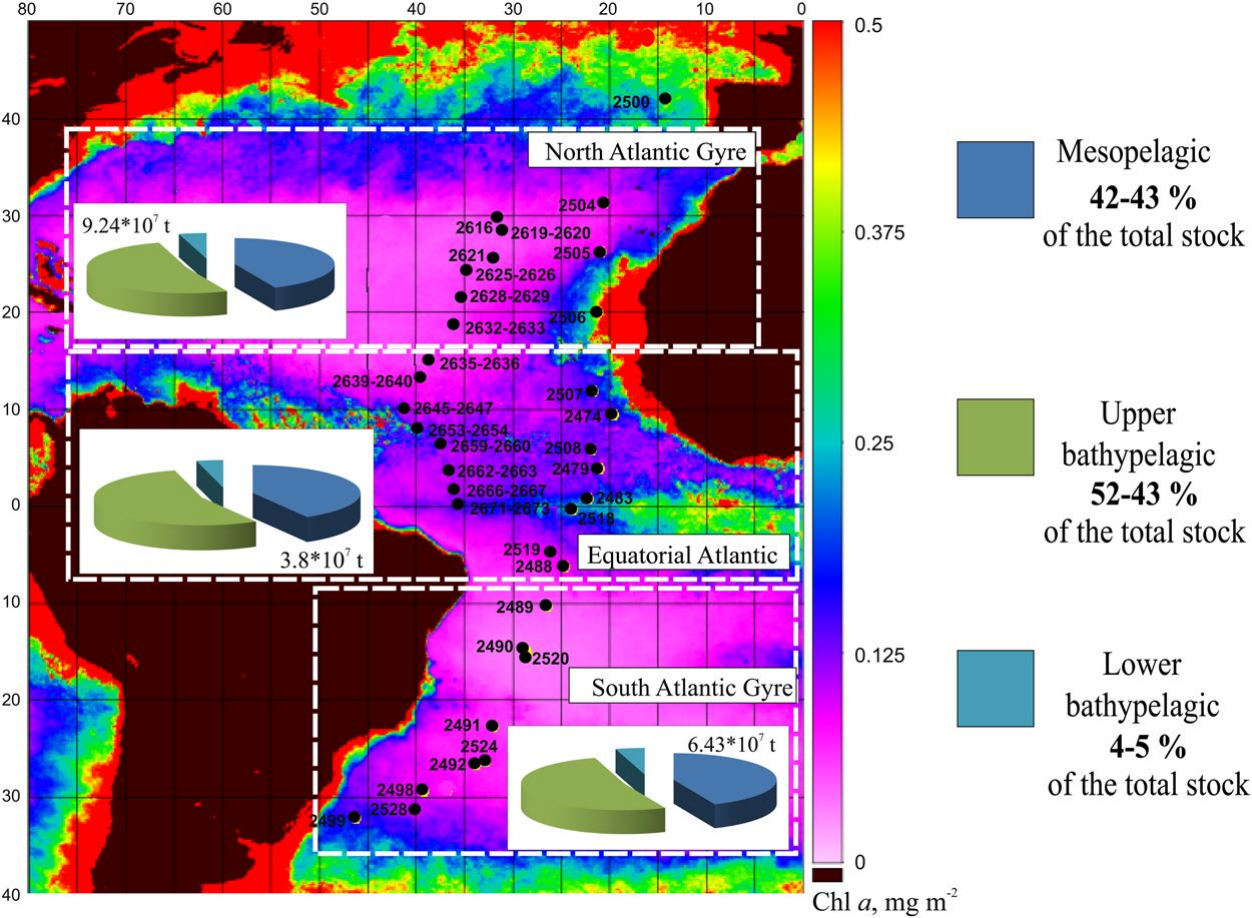
Stations (black circles) sampled during the cruises of R/V “Akademik Sergey Vavilov” (see also Table 1), with assessed standing stock (wet weight biomass) of deep-pelagic shrimps and contributions (%) of the vertical depth strata to the total stocks in the North, Equatorial, and South Atlantic. Data on lower mesopelagic should be considered with caution, as regressions are not statistically robust (p = 0.089). Background: surface chlorophyll a concentration averaged over 2013; scale (mg m^−2^) is given on the right.

Samples were collected during three cruises of the R/V “*Akademik Sergey Vavilov*” in 2013–2016 (Fig. 1, Table 1). As in previous studies^12,13^, we minimized the land and the seafloor effects, conducting the survey at a distance at least hundreds of meters from the bottom and hundreds of kilometers from the nearest landmass. In particular, we excluded benthopelagic species, which can form over 50% of the total plankton biomass close to the seafloor or continental slopes and seamounts^17^.

**Table 1:** List of stations (R/V “*Akademik Sergey Vavilov*”) from which assessments of the shrimp biomass were based.

Station No.	Date	Latitude	Longitude	Sampling zones	Surface chlorophyll-a concentration (mg m^−2^)	Depth (m)
2474	24.10.2012	9°25′N	19°44′W	EMUL	0.12	4282
2479	25.10. 2012	3°51′N	21°15′W	EMUL	0.13	5235
2483	28.10. 2012	0°50′N	22°26′W	EMUL	0.17	4360
2488	29.10. 2012	6°12′S	24°05′W	EMU	0.09	3800
2489	30.10. 2012	10°18′S	26°37′W	EMUL	0.05	5500
2490	01.11. 2012	15°06′S	28°45′W	EMUL	0.03	5030
2491	03.11. 2012	22°43′S	32°05′W	EMUL	0.07	4690
2492	05.11. 2012	26°39′S	33°58′W	EMUL	0.07	4710
2498	07.11. 2012	29°27′S	39°15′W	EMUL	0.09	4724
2499	10.11. 2012	32°11′S	46°26′W	T	0.10	3780
2500	23.09. 2013	41°58′N	14°17′W	EMUL	0.29	5000
2504	27.09. 2013	31°12′N	20°48′W	EMU	0.09	3150
2505	29.09. 2013	26°14′N	21°03′W	EMUL	0.12	4700
2506	30.09. 2013	19°59′N	21°22′W	EMUL	0.46	3780
2507	03.10. 2013	11°50′N	21°47′W	EMUL	0.17	4900
2508	04.10. 2013	5°50′N	22°00′W	EMUL	0.12	3800
2518	10.10. 2013	1°25′S	24°00′W	EMUL	0.17	4700
2519	11.10. 2013	07°01′S	26°04′W	EMUL	0.16	4500
2520	14.10. 2013	15°35′S	28°41′W	EMUL	0.03	5100
2524	19.10. 2013	26°23′S	32°53′W	EMU	0.07	3000
2528	21.10. 2013	31°00′S	40°38′W	EMU	0.09	2250
2616	12.10.2016	30°02.5N	32°11.8	EMUL	0.09	5244
2619	13.10.2016	29°06N	32°54.95W	EMUL	0.08	4412
2620	13.10.2016	29°6N	32°54.95W	EMUL	0.08	4771
2621	14.10.2016	26°34.5N	33°57W	EMUL	0.08	5029
2625	15.01.2016	24°09.8N	34°58.5W	EMUL	0.08	5364
2626	15.01.2016	24°08.9N	34°58.4W	EMUL	0.08	5364
2628	16.10.2016	22°05.6N	35°50.9W	EMUL	0.08	5354
2629	16.10.2016	22°05.6N	35°50.9W	EMUL	0.08	5354
2632	17.10.2016	19°34.7N	36°56.2W	EMUL	0.08	5548
2633	17.10.2016	19°34.7N	36°56.2W	EMUL	0.08	5548
2635	18.10.2016	16°37.8N	38°13.8W	EMUL	0.09	5207
2636	18.10.2016	16°37.8N	38°13.8W	EMUL	0.09	5207
2639	19.10.2016	14°07.1N	39°31.1W	EMUL	0.10	4816
2640	19.10.2016	14°07.1N	39°31.1W	EMUL	0.10	4816
2645	21.10.2016	10°49N	41°00.5W	EMUL	0.19	4206
2647	21.10.2016	10°47.4N	41°00.8W	EMUL	0.19	4618
2653	23.10.2016	8°16.6N	38°25.2W	EMUL	0.20	3941
2654	23.10.2016	8°16.6N	38°25.2W	EMU	0.20	3941
2659	24.10.2016	7°11.7N	37°50W	EMUL	0.20	3572
2660	24.10.2016	7°11.7N	37°50W	EMUL	0.20	3572
2662	25.10.2016	4°46.3N	37°11.1W	EMUT	0.18	4646
2663	25.10.2016	4°46.3N	37°11.1W	EMUL	0.18	4646
2666	26.10.2016	2°09.6N	36°32.8W	EMUL	0.15	4038
2667	26.10.2016	2°09.6N	36°32.8W	EMUL	0.15	4038
2671	27.10.2016	0°00.3N	36°00.0W	EMUL	0.16	4515
2672	27.10.2016	0°00.3N	36°00.0W	EMU	0.16	4515
2673	27.10.2016	0°00.3N	36°00.0W	T	0.16	4515

Date = day.month.year. Sampling zones: E - epipelagic, M - main thermocline (mesopelagic), U- upper bathypelagic, L - lower bathypelagic; T - total water column (0–3000 m oblique, net was not closed). Depth = bottom depth.

Sampling was conducted using a closing Bogorov-Rass (BR) plankton net (1-m^2^ opening, 500-μm mesh size, towed vertically at a speed of 1 m s^−1^). This gear has proven successful for sampling deep-sea mesozooplankton of the size range of 1–50 mm^9–11^ as well as micronektonic shrimps^12,13^. Vertical nets allow depth to be efficiently metered by wire out; wire angle was always <5^o^ from perpendicular, resulting in a vertical accuracy 0.4% (measured and estimated net depth differed by 12 m at 3000 m depth).

### Comparison of oblique and vertical sampling

We compared collection efficiency of oblique and vertical sampling at night within the depth range 0–800 m, roughly covering the epi- and mesopelagic zones. The BR net was deployed to a depth of 800 m, then opened and towed vertically upwards, and finally closed at 200 m with a mechanical device, thus sampling the mesopelagic. The next deployment, 7–10 min later, sampled the epipelagic (0–200 m). Results from both hauls were then integrated. In some cases we sampled the layer 0–800 m at once (Table 2, last two rows). Each nighttime net sampling event was conducted after sunset between 18:00 and 01:00 (next day).

**Table 2:** Comparison of pelagic shrimp abundance and biomass estimates generated from vertical net and oblique trawl sampling in the epi- and mesopelagic at night.

Station number		Latitude		Longitude		Date		Time		Number caught (raw)		Wet weight biomass (g)		Standardized abundances (no. 10^−3^ m^−3^)		Standardized biomass (g 10^−3^ m^−3^)	
Net	Trawl	Net	Trawl	Net	Trawl	Net	Trawl	Net	Trawl	Net	Trawl	Net	Trawl	Net	Trawl	Net	Trawl
2616	2618	30°02.5N	30°00.8–29°56.3N	32°11.8W	32°11'1–32°11'2W	13 Oct 2016	13 Oct 2016	00:10–01:00	01:20–03:00	3	16	0.12	9.35	3.33	0.33	0.13	0.19
2629	2631	22°05.6N	22°02.6–21°58.0N	35°50.9W	35°52.2–35°53.7W	16 Oct 2016	16–17 Oct 2016	21:40–22:30	23:50–01:06	2	23	0.71	12.91	2.22	0.48	0.79	0.27
2636	2637	16°37.8N	16°37.8–16°34.1N	38°13.8W	38°14.9–38°16.6W	18 Oct 2016	18 Oct 2016	20:30–21:20	22:17–23:40	5	23	21.7	13.76	5.56	0.48	24.06	0.29
2647	2649	10°47.4N	10°46.0–10°43.6N	41°00.8W	41°01.2–41°05.3W	22 Oct 2016	22 Oct 2016	20:00–20:50	21:15–22:33	5	49	2.7	23.34	5.56	1.02	3.00	0.49
2654	2656	8°16.6N	8°13.9–8°09.2N	38°25.2W	38°24.0–38°24.0W	23 Oct 2016	24 Oct 2016	21:00–21:50	02:00–03:30	1	47	2.1	27.35	1.11	0.98	2.33	0.57
2662	2665	4°46.3N	4°43.5–4°39.3N	37°11.1W	37°08.7–37°07.8W	24 Oct 2016	25–26 Oct 2016	18:00–18:50	22:50–00:19	1	34	3.9	13.15	1.11	0.71	4.33	0.27
2667	2669	2°09.6N	2°02.5–2°03.8N	36°32.8W	36°32.3–36°31.0W	26 Oct 2016	26–27 Oct 2016	21:10–22:05	23:04–00:39	1	40	15.5	12.5	1.11	0.83	17.22	0.26
2672	2675	0°00.3N	0°01.0–0°03.7S	36°00.0W	36°01.1–35°59.1W	27 Oct 2016	27–28 Oct 2016	18:00–18:45	22:49–00:47	3	70	13.4	29.8	3.33	1.46	14.89	0.62
2673	2675	0°00.3N	0°01.0–0°03.7S	36°00.0W	36°01.1–35°59.1W	27 Oct 2016	27–28 Oct 2016	20:15–20:30	22:49–00:47	3	70	13.5	29.8	3.33	1.46	15.00	0.62
2674	2675	0°00.3N	0°01.0–0°03.7S	36°00.0W	36°01.1–35°59.1W	27 Oct 2016	27–28 Oct 2016	20:40–20:55	22:49–00:47	2	70	6.44	29.8	2.22	1.46	7.16	0.62

Multiple vertical net samples at 0°00.3N (stations 2672–2674) were compared to an oblique trawl sample 2675 from the same site.

The vertical net sampling was followed by oblique trawl hauls (Table 2). We used a non-closing Isaacs-Kidd midwater trawl: 5.5-m^2^ opening, 5-mm mesh size, with a smaller-mesh net (0.18-m^2^ opening, 1-mm mesh size) forming the back end of the trawl. The trawl was deployed to a depth of 800 m and then slowly retrieved with a vertical speed 10–11 m/min (vessel speed ranged between 2 and 3 knots). The distance sampled was assessed by GPS/GLONASS with a precision of about 20 m (0.2% over the trawl path of ~8800 m). The total volume of water filtered ranged between 46 and 50 × 10^3^ m^3^. Each trawl sampling lasted 1.25–2 h between 21:15 and 03:30 h (next day). Since the mesh size differed between the two nets (5 mm for the IKMT vs. 0.5 mm for the BR), shrimps <30 mm total length (larvae and juveniles) were removed from the analyses. All decapod specimens were in good condition, with all appendages present in catches from both gears. At one site we collected three vertical net samples (St. 2672–2674) and one oblique trawl sample (St. 2675) (Table 2). In Analysis 1, we compared each of the BR samples with the same IKMT trawl, thus having three comparisons at this site. In Analysis 2, we used a mean for the three BR nets compared to the IKMT net for a single comparison.

### Comparison of day and night samples

**Figure 2:**
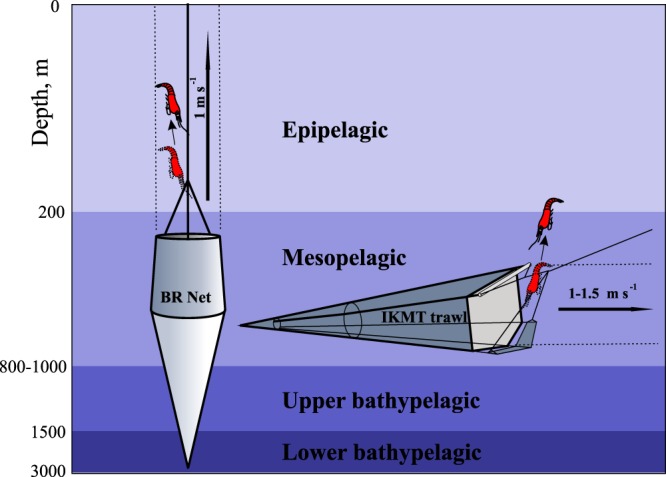
Comparison of vertical Bogorov-Rass (BR) net and oblique trawl sampling for deep-pelagic shrimps, with possible escapement trajectories.

We compared 13 day/night sample pairs collected by the BR net (Table 3). Samples were taken between 1 h after sunset and 1 h before sunrise (night samples) and between 1 h after sunrise and 1 h before sunset (day samples) in order to avoid the confounding effects of diel vertical migration. During each set we consecutively sampled four discrete-depth strata (Fig. 2): (1) the epipelagic zone (0–200 m); (2) the main thermocline (from 200 m to the depth of the 7 °C isotherm, usually within 800–1000 m), which we consider here to represent the mesopelagic zone; (3) the zone from the lower boundary of the main thermocline to 1500 m, mainly Antarctic Transitional Water, which we define as the upper bathypelagic; and (4) the layer 1500–3000 m, mainly North Atlantic Deep Water, which we define as the lower bathypelagic^12,13^. As our sampling was associated with water masses, the boundary between the meso- and bathypelagic zones as defined here did not always coincide with the traditional one (1000 m).

**Table 3:** Comparison of day and night net samples (13 pairs, each taken at the same site, night (N) and day (D)).

Station Numbers		Abundances (ind. per 1000 m^−3^) within various depth ranges (m)										Biomass (mg per m^−3^) within various depth ranges (m)									
		0–3000		0–200		200–800		800–1500		1500–3000		0–3000		0–200		200–800		800–1500		1500–3000	
N	D	N	D	N	D	N	D	N	D	N	D	N	D	N	D	N	D	N	D	N	D
2620	2619	0.67	0.33	0.00	0.00	2.50	2.50	0.00	0.00	0.67	0.00	0.88	0.57	0.00	0.00	6.50	4.25	0.00	0.00	0.03	0.00
2622	2621	0.67	0.33	0.00	0.00	0.00	0.00	1.43	0.00	0.67	0.67	0.04	0.02	0.00	0.00	0.00	0.00	0.00	0.00	0.04	0.04
2626	2625	0.67	0.67	5.00	0.00	2.50	5.00	0.00	0.00	0.00	0.00	0.47	0.03	1.50	0.00	2.75	0.20	0.00	0.00	0.00	0.00
2629	2628	1.00	0.00	5.00	0.00	2.50	0.00	1.43	0.00	0.00	0.00	0.28	0.00	0.50	0.00	1.75	0.00	0.07	0.00	0.00	0.00
2633	2632	0.67	0.33	0.00	0.00	5.00	2.50	0.00	0.00	0.00	0.00	3.55	0.10	0.00	0.00	26.50	0.75	0.00	0.00	0.00	0.00
2636	2635	2.00	1.00	0.00	0.00	12.50	7.50	1.43	0.00	0.00	0.00	7.25	1.17	0.00	0.00	54.03	8.75	0.21	0.00	0.00	0.00
2640	2639	0.67	1.67	5.00	0.00	2.50	10.00	0.00	1.43	0.00	0.00	4.15	0.22	0.25	0.00	31.00	1.50	0.00	0.07	0.00	0.00
2647	2645	1.33	3.00	0.00	0.00	5.00	10.00	2.86	5.71	0.00	0.67	4.12	25.97	0.00	0.00	6.75	3.00	13.79	84.57	0.00	11.67
2654	2653	1.00	1.67	0.00	0.00	2.50	10.00	2.86	1.43	0.00	0.00	3.00	6.43	0.00	0.00	5.25	30.00	9.86	10.43	0.00	0.00
2660	2659	2.33	2.67	0.00	0.00	12.50	12.50	1.43	2.86	0.67	0.67	5.32	4.72	0.00	0.00	37.75	14.50	0.07	10.93	0.53	0.47
2663	2662	0.67	0.67	0.00	0.00	0.00	2.50	2.86	0.00	0.00	0.00	6.67	0.97	0.00	0.00	0.00	0.25	28.57	0.00	0.00	0.00
2667	2666	0.33	0.67	0.00	0.00	2.50	0.00	0.00	1.43	0.00	0.67	5.17	5.65	0.00	0.00	38.75	0.00	0.00	24.14	0.00	0.03
2672	2671	2.00	1.33	0.00	0.00	7.50	5.00	2.86	1.43	0.67	0.67	19.13	2.99	0.00	0.00	33.50	3.45	52.14	0.11	5.00	5.00
Wilcoxon-Mann-Whitney test		0.713		0.078		0.732		0.424		0.709		0.182		**0**.**037**		**0**.**045**		0.826		0.650	
Kolmogorov-Smirnov test		0.828		0.828		0.995		0.995		0.709		0.226		0.489		**0**.**087**		1.000		1.000	

Bold boxes indicate a statistically significant difference between day and night samples (*p* < 0.05 for Wilcoxon-Mann-Whitney test and *p* < 0.10 for Kolmogorov-Smirnov test).

### Identification and assessment of the shrimp abundance and biomass

All shrimps were identified to species using Chace^18^ for Oplophoridae and Acanthephyridae, Vereshchaka^8^ for Benthesicymidae, and Vereshchaka^19,20^ for Sergestidae. Synonymy of species was corrected according to WoRMS^21^. Specimens were measured and weighed to within 0.1 mm and 0.05 g, respectively.

For the assessment of shrimp biomass we used prior results of multivariative analyses^12,13^, which showed that depth layer and surface chlorophyll-*a* concentration (Chl) are the two main factors affecting the standing stock biomass of the main plankton groups plus shrimps. Chl, derived from satellite imaging, was used as a proxy of surface productivity. Chl *a* data were taken from Aqua MODIS (level 3, 4-km resolution, https://oceancolor.gsfc.nasa.gov/) and averaged over one year preceding the sampling date and over a 5° × 5° rectangle (with the sampling site in the center).

The total shrimp biomass over large oceanic areas was estimated as B = ∑ B_*i*_ × *S*_*i*_, where B is shrimp biomass density within a depth layer or in the whole water column over a selected area, *i* is a Chl range varying from 0.002 to 0.498 mg × m^−2^, with a step of 0.002 mg × m^−2^, B_*i*_, and *S*_*i*_ are biomass density of the range *i* and the area occupied by these values, respectively. B_*i*_ were assessed from equations lg(B_*i*_) = *a* × lg(Chl_*i*_) + *b*, where *a* and *b* are retrieved coefficients of regression.

Calculations, statistical procedures, regression analysis, and ANOVA tests were carried out with the use of STATISTICA and PAST 3.04^22^. Comparison of oblique/vertical sampling and day/night sampling was based on restricted number of data, which did not pass normality tests. In this case we used nonparametric tests: Mann-Whitney test for equal medians and Kolmogorov-Smirnov test for equal distributions. Regressions between Chl and biomass were based on a significantly richer dataset, in which variables (if log-transformed) passed the test of normality and could be analyzed quantitatively. We further made a regression analysis using the Linear Bivariate model and the Ordinary Least Squares algorithm, which allowed assessment of a 95% confidence band for the fitted line (not for the data points). During the log-transformation of the zero biomass values, we added half an individual per haul for abundance densities and half of the minimal individual weight (0.05 g) for biomass densities.

Flowmeters were not used, so volume filtered per tow was calculated geometrically (mouth area × distance through water). Net filtration coefficients were set to 1 for both BR nets and Isaacs-Kidd trawls, with the proviso that the actual values may be slightly different: around 1 for a vertical plankton net^23^ and 0.92 for our modification of the trawl^24^.

## Results

### Faunal composition

In oblique and vertical samples 38 shrimp species belonging to four families were recorded, broken down as follows:

- Acanthephyridae (16 species): *Acanthephyra acanthitelsonis*, *A*. *acutifrons*, *A*. *brevirostris*, *A*. *cucullata*, *A*. *curtirostris*, *A*. *eximia*, *A*. *kingsleyi*, *A*. *pelagica*, *A*. *purpurea*, *A*. *quadrispinosa*, *Hymenodora gracilis*, *Meningodora marptocheles*, *M*. *mollis*, *Notostomus auriculatus*, *N*. *elegans*, and *N*. *gibbosus*.
- Oplophoridae (4 species): *Oplophorus spinosus*, *Systellaspis braueri*, *S*. *cristata*, and *S*. *debilis*.
- Benthesicymidae (8 species): *Bentheogennema intermedia*, *Gennadas bouvieri*, *G*. *brevirostris*, *G*. *capensis*, *G*. *elegans*, *G*. *gilchristi*, *G*. *scutatus*, and *G*. *talismani*.
- Sergestidae (10 species): *Allosergestes pectinatus*, *A*. *sargassi*, *Deosergestes henseni*, D. *corniculum*, *Gardinerosergia splendens*, *Neosergestes edwardsi*, *Parasergestes armatus*, *P*. *vigilax*, *Phorcosergia wolffi*, and *Robustosergia extenuata*.

The following species were dominant:

- The North Atlantic Gyre: *Acanthephyra purpurea* and *Systellaspis debilis* (mesopelagic), *Acanthephyra acanthitelsonis* (upper bathypelagic).
- Equatorial Atlantic: *Acanthephyra acanthitelsonis*, *Acanthephyra kingsleyi*, *Gennadas talismani*, and *Notostomus elegans* (mesopelagic) and *Notostomus gibbosus* (upper bathypelagic).
- The South Atlantic Gyre: *Acanthephyra quadrispinosa* (mesopelagic).

In all areas, *Acanthephyra brevirostris* and *Hymenodora gracilis* dominated in the lower bathypelagic.

### Comparison of oblique and vertical sampling

Raw counts and standardized values of shrimp abundance and biomass are presented in Table 2. Analysis 1 (each of the BR samples taken at stations 2672–2674 compared with the same IKMT trawl independently) and Analysis 2 (a mean for the three BR nets compared to the IKMT net for a single comparison) retrieved similar results and showed that vertical nets provide significantly higher abundance and biomass estimates than trawls. Indeed, in both cases non-parametric Wilcoxon — Mann — Whitney (WMW) and Kolmogorov-Smirnov (KS) tests showed that abundance and biomass estimates from vertical net and oblique trawl samples were significantly different. Analysis 1 retrieved *p* = 0.002 for abundances (both WMW and KS) and *p* = 0.003 (WMW) and 0.0002 (KS) for biomass, while Analysis 2 retrieved *p* = 0.003 (WMW) and 0.001 (KS) for abundances and *p* = 0.014 (WMW) and 0.001 (KS) for biomass. Vertical net sample estimates were higher both for abundance (4.2 ± 3.8 times higher in Analysis 1 and 4.7 ± 1.5 times higher in Analysis 2) and biomass (23.9 ± 9.0 times higher in Analysis 1 and 24.9 ± 11.2 times higher in Analysis 2) (values are given in the format Mean ± SD).

### Comparison of day and night samples

Comparison of day/night vertical net series revealed depth-specific differences in abundance and biomass. There was no statistically significant difference in abundance at any depth stratum and in biomass in the bathypelagic and in the whole water column (Table 3). However, biomass values significantly differed in the epipelagic (0–200 m, WMW test, *p* = 0.037) and in the mesopelagic (0–800 m, WMW tests, *p* = 0.045; KS test, *p* = 0.087) as a function of time of day (Table 3). In the mesopelagic, where biomass values differed, night biomass (B_N_) was generally higher than day biomass (B_D_). In order to estimate an average difference, we calculated the ratio B_N_/B_D_ for each pair of day/night sample. Using this approach, nighttime mesopelagic samples were 38.4 ± 29.2 times larger with respect to biomass than day samples. B_N_/B_D_ ratio for the epipelagic was impossible to estimate, as no decapod was caught in this layer in the daytime.

### Regressions

**Figure 3:**
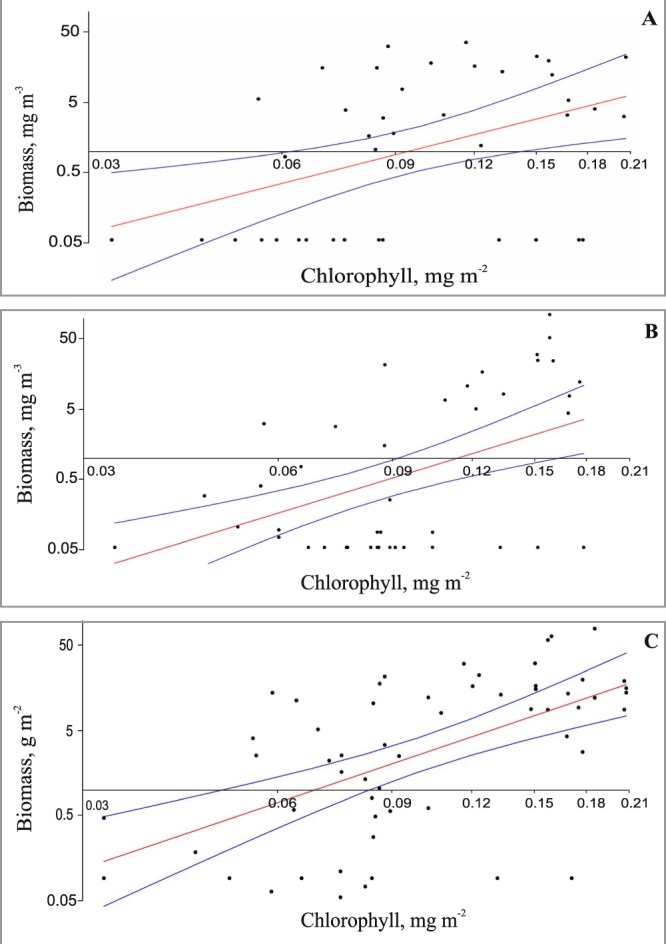
Regressions (Linear model, Ordinary Least Squares algorithm) showing relationship of shrimp biomass with average surface chlorophyll a concentration: (**A**) mesopelagic, (**B**) upper bathypelagic, (**C**) whole water column. Red – regression lines, blue – 95% confidence band for the fitted line (not for the data points).

Both log-transformed shrimp biomass estimates from net samples and averaged Chl *a* values passed tests for normality and were highly correlated (Table 4). Moreover, in all depth strata except the epipelagic, in which the shrimp biomass was minimal, the shrimp standing stock was also correlated with Chl *a* (Fig. 3). The regressions were robust for the whole water column, the mesopelagic, and the upper bathypelagic (*p* ≤ 0.01). For lower bathypelagic, regressions were less statistically significant (p < 0.1). Overall, extension of the previous dataset (p > 0.01–0.05 in Vereshchaka *et al*., 2016 – Table 3) has resulted in more robust regressions. Coefficients of determination (R^2^) were also significantly higher (with an exception of the lower bathypelagic - Table 4).

**Table 4:** Regressions between surface chlorophyll *a* concentration (Chl, mg m^−2^) and shrimp biomass (B, g m^−2^ for the whole water column and mg m^−3^ for vertical zones): statistical significance (p), equations, and сoefficients of determination (R^2^).

Vertical zones	p	Regression equations	95% bootstrapped confidence interval for slope (N = 1999)	R^2^	R^2^, previous data^12^	Number of samples	Number of samples, previous data^14^
Epipelagic	0.489	n/a	n/a	n/a	Test for normal distribution not passed	41	35
Main thermocline	0.004	lg(B) = 2.34 lg(Chl) + 2.37	1.14–3.69	0.197	0.07	41	35
Upper bathypelagic	0.00005	lg(B) = 2.88 lg(Chl) + 2.75	1.70–4.15	0.280	0.13	53	35
Lower bathypelagic	0.089	lg(B) = 1.02 lg(Chl) + 0.19	−0.25–2.03	0.067	0.11	46	26
Whole water column	0.000003	lg(B) = 2.56 lg(Chl) + 3.00	1.73–3.37	0.332	0.07	57	36

Data on lower mesopelagic should be considered with caution, as regressions are not statistically robust (*p* = 0.089).

### Deep-pelagic shrimp biomass standing stocks

Having found statistically significant regressions between shrimp biomass estimates generated from net sampling and Chl *a*, we calculated the total shrimp standing stock (biomass under 1 m^2^ in the water column from 0–3000 m) and standing stocks within the meso-, upper bathy-, and lower bathypelagic strata (biomass under 1 km^2^, integrated over each of these layers). As the regressions for the lower bathypelagic were not robust, further assessments for this zone are given for illustrative purposes and should be considered cautiously. This process was iterated for three rectangular areas roughly corresponding to the North and South Atlantic Gyres and to the Equatorial Atlantic (Fig. 1). Higher standing stocks were found in the North Atlantic Gyre and Equatorial Atlantic compared to the South Atlantic Gyre (Table 5). Equatorial waters were the richest in shrimp biomass, roughly twice that of either gyre (Table 5).

**Table 5:** Assessment of the total and average (per square kilometer) standing stock biomass of deep-pelagic shrimps of major depth zones and of the whole water column over selected geographic areas in the Atlantic Ocean (as indicated in Fig. 1).

	North Gyre					Equatorial Waters					South Gyre				
	2014	2015	2016	Avg.	SD	2014	2015	2016	Avg.	SD	2014	2015	2016	Avg.	SD
**Estimated total standing stock in the depth zone (**×**10**^**7**^ **t)**															
Mesopelagic	2.01	2.27	2.10	2.13	0.08	1.77	1.86	1.95	1.86	0.05	1.52	1.48	1.46	1.49	0.02
Upper bathypelagic	2.39	2.79	2.51	2.56	0.12	2.28	2.44	2.56	2.43	0.08	1.84	1.77	1.72	1.78	0.03
Lower bathypelagic	0.21	0.23	0.22	0.22	0.01	0.17	0.18	0.18	0.18	0.00	0.16	0.16	0.15	0.16	0.00
Water column	8.70	9.92	9.09	9.24	0.36	7.87	8.31	8.69	8.29	0.24	6.6	6.42	6.28	6.43	0.09
**Estimated average density in the area (t km** ^**−2**^ **)**															
Mesopelagic	1.14	1.28	1.17	1.20	0.06	2.03	2.19	2.18	2.13	0.07	1.02	1.00	0.99	1.00	0.01
Upper bathypelagic	1.36	1.57	1.40	1.44	0.09	2.61	2.87	2.87	2.78	0.12	1.23	1.19	1.17	1.20	0.02
Lower bathypelagic	0.12	0.13	0.12	0.12	0.00	0.20	0.21	0.21	0.21	0.00	0.11	0.10	0.10	0.10	0.00
Water column	4.95	5.59	5.05	5.20	0.28	9.02	9.78	9.74	9.51	0.35	4.41	4.32	4.26	4.33	0.06

Assessments were made for 2014, 2015, and 2016 years separately, average values (Avg.) and standard deviations (SD) for 2014–2016 presented. Owing to different regressions, assessed biomass in the whole water column exceeds sum of estimated biomasses of all depth zones.

The contribution of various depth strata to the total water-column standing stock was similar in the three selected areas: the upper bathypelagic zone and the main thermocline zone contributed the largest component of the shrimp biomass (52–54% and 42–43%, respectively), while the lower bathypelagic contributed 4–5%.

In order to assess possible interannual variation, we retrieved data of the average Chl *a* concentrations from the same areas for 2014 and 2015 and found that the resulting fluctuations of the assessed shrimp biomass were not significant (Table 5), thus suggesting that the total shrimp standing stock does not greatly change on timescales of a few years.

## Discussion

Comparison of sampling methods suggests that vertically towed nets such as the Bogorov-Rass are more accurate for estimating deep-pelagic shrimp biomass than horizontally or obliquely towed nets. One cannot catch more than is present in the water column, only less (due to avoidance and escapement through mesh extrusion). Therefore, that gear which yields the highest catch per sampled volume would be considered the most efficient. Comparisons with obliquely towed trawl data obtained at the same place immediately after net tows showed that vertical nets provide higher abundance and biomass estimates. Since the differences are statistically robust, we suggest that vertical nets minimize avoidance of shrimps, which usually escape in a vertical direction (Fig. 2). As vertical nets result in greater biomass estimates (23.9 ± 9.0 times higher), previous assessments of the deep-pelagic shrimp biomass based solely on horizontal and oblique trawl data should be reconsidered. Recent findings^12^ show that pelagic decapods may contribute 50% of the total net plankton/micronekton zooplankton standing crop in the Tropical Atlantic. If this effect is panoceanic, previous estimations of the total plankton/micronekton biomass in the deep should also be reviewed.

Comparison of day and night net samples shows that the biomass estimations in the epi- and mesopelagic are strongly dependent on the time of day. Our data show that in the mesopelagic night values are 38.4 ± 29.2 times higher than day values (respective values for the epipelagic is likely even higher but cannot be quantified, as no decapod was caught in this layer in the daytime). This effect may be owing to two causes. Firstly, many species perform diel vertical migrations and aggregate in the epi- and upper mesopelagic at night and migrate into deeper layers by day. Secondly, visual escapement is more efficient in illuminated (epipelagic) and twilight (mesopelagic^25^) zones. Further, comparison of day/night vertical net series did not reveal significant differences in abundance between night and day catches. Summary of both trends (abundance and biomass) would seem to suggest that net samples caught slightly more animals at night (but not enough to make for a significant difference), but that the extra animals were much larger (hence more biomass, which is approximately cubic function of linear size). Assessments of shrimp standing stocks in the deeper layers under the main thermocline (both upper and lower bathypelagic) do not appear to significantly depend on the time of day. Probably, light conditions in this zone are similar at night and in the daytime, thus making results comparable. More important and unexpected is invariance (to the time of day) of the whole water column biomass. Part of the shrimp assemblages occur in the dimly illuminated main thermocline and hypothetically could escape from gear more efficiently during daylight, making the night total biomass estimate larger than that of daytime. This effect was observed in our catches in some cases but was not statistically supported (Table 3). The invariance of the whole water column biomass to the time of day greatly facilitates biomass assessment surveys in the future, making them less dependent on the time of day of sampling as long as sampling is conducted well into the bathypelagic zone.

Pelagic decapods feed mainly on pelagic copepods, although euphausiids, chaetognaths, and fishes may also be significant components of their diet^3,4,8,26,27^. The decapods are thus second-level consumers, do not utilize primary production directly, and regression between their biomass and surface Chl *a* concentration are expected to be not as robust as for the mesoplankton, which encompasses mainly first-level consumers. Our data, however, did show statistically significant regressions between the averaged surface Chl *a* values and shrimp biomass in all depth layers excepting the epipelagic. Preliminary results have shown the existence of such regressions^12^ (Table 3), and incorporation of new data into the previous dataset resulted in much more robust regressions (Table 4, Fig. 3). With future enrichment of the existing dataset, including bathypelagic and vertical net sampling in other areas of the World Ocean, regressions that are even more refined are expected.

It is noteworthy that in all robust regressions (mesopelagic, upper bathypelagic, water column) the slope values are higher that 1 (Table 4, *p* < 0.05). This difference suggests that the decapods take a larger share of primary production in more productive areas. Indeed, in the low production areas of the Atlantic anticyclonic gyres the structure of mesoplanktonic communities is more complex and the number of species is higher than in more productive Atlantic areas^9,10,13^. According to general ecological concepts^28^, in these areas communities utilize primary production more efficiently owing to an increased number of ecological niches, which results in a smaller proportion of shrimps and a larger proportion of other planktonic consumers. In more productive areas the number of species and ecological niches decreases and the shrimps may take a larger share of primary production.

The assessed values of shrimp biomass revealed the highest average total shrimp standing stock biomass in the low latitude Atlantic to be Equatorial waters, owing to increased surface productivity near Equatorial Divergence: 9.5 t km^−2^ versus 5.2 t km^−2^ and 4.3 t km^−2^ in the North and South Gyres, respectively. The total shrimp standing stock, however, was higher in the North Atlantic Gyre (92.4 million t) because of its larger area, followed by the Equatorial (82.9 million t) and South Atlantic Gyre (64.3 million t). Integrating these estimates, the total shrimp crop between 40°N and 40°S may now roughly be assessed to be 240 million t. The previous estimates of the shrimp standing stock in the same area were 5–19 million t (i.e. 50% of total net zooplankton/micronekton biomass, estimated^12^ at 10–38 million t), which is one order of magnitude lower than assessments presented here.

The bulk (52–54%) of shrimp biomass was concentrated from the lower border of the main thermocline (800–1000 m) to a depth of 1500 m (Fig. 1), in the layer designated as the upper bathypelagic^12,13^. The mesopelagic harbored a lesser, but still important, part of the total shrimp biomass (42–43%). Both zones encompass the vertical range of the diel vertical migrations of most pelagic shrimps^3,4^, where night feeding in upper productive layers and hiding from predators in deeper, darker waters by day provide the optimal conditions for deep-pelagic shrimps.

The depth zone 200–1500 m accounted for 95–96% of the total shrimp standing crop. The lower bathypelagic is a zone where vertical migrations of shrimps are nearly absent and the concentration of potential plankton food drastically decreases^13,29^. In spite of a very extensive vertical range (from 1500 to 3000 m), which is nearly half of the total water column, the lower bathypelagic harbored only 4–5% of the total shrimp crop. Owing to such a small contribution, our assessments of the shrimp stock in this zone, which are not as accurate as in other layers, should not significantly misrepresent the global stock values.

Our results mirror similar findings concerning mesopelagic fish biomass related to their efficient trawl avoidance^16,30,31^. Namely, a new methodology (acoustics for fishes, vertical nets for crustaceans) yields new insights into global biomass. Our data suggest the shrimp crop is about 240 million t for the Atlantic between the 40-degree latitudes (Fig. 1, Table 5). The survey area in this study was ~51.46 million km^2^, i.e. 14% of the World Ocean area, and direct extrapolation on the global scale gives 1700 million t of deep-pelagic shrimp biomass on Earth. We expressly note here that this extrapolation derives from a very restricted (on the global scale) material collected in the severely underexplored meso-to-bathypelagic domain^32^. That said, these data, which are among the most robust of their kind, suggest that further research is sorely need to fill this massive data gap, and that further research in this vein will corroborate these results. Previous extrapolations, derived from trawl data, for the Atlantic^3,4^, Indian^7^, and South-East Pacific Oceans^8^ range roughly between 0.1–0.5 mg m^−3^ for the top 1000 m of the water column (i.e., 0.1–0.5 t km^−2^). It is possible that these estimates are at least two orders of magnitude too low, driving the need for more detailed quantitative assessments.

Our results also call for reevaluation of biological pump, which includes the passive sinking of particulate organic matter, diffusion and advection of dissolved organic matter, and active transport by the vertical migration of animals^33^. The importance of active transport of carbon involving the transfer of organic matter consumed by plankton in the epipelagic at night to their daytime residence depths through a combination of respiration, excretion, defecation, and mortality has been recently recognized^34–37^. As pelagic decapods are an abundant and important component of the pelagic communities^38–40^, their contribution to active carbon flux are expected to be significant. Indeed, the latest estimations of total active downward carbon flux caused by pelagic decapods range from 383 to 625 mg C m^−2^ day^−1^ in the North Pacific Subtropical Gyre^41^. These values were equal to 2.1–8.8% of passive flux in the mesopelagic (depth 262–711 m) or to 1.5–2.4% of passive flux at the base of the euphotic zone (depth 173 m). Since these results were based on trawl samples, actual contribution of pelagic decapods may be greatly higher and comparable to the passive flux.

All global estimates to date, including those presented here, should be considered extremely preliminary. Regressions in specific ocean basins depend on local trophic interactions, whose transfer efficiency may vary several orders of magnitude, from less than 0.001 to significantly >0.1, even in oligotrophic waters^42^. In temperate, subpolar, and polar waters regressions between surface Chl *a* and deep-pelagic shrimp biomass may significantly differ from those in tropical areas. Like fishes^16^, deep-sea shrimps may be even more abundant in higher latitudes. If direct extrapolation of our data on a global scale provides a correct order of magnitude (retrieved value 1700 million tons), the shrimp stock is comparable to the lower range of fish biomass estimates, falling between the estimates of ~1000 million tons^14,15^ and 11000–15000 million tons^16^. Deep-sea pelagic shrimp may thus be comparable to fishes with respect to their role in global marine processes. Most pelagic shrimps migrate over an extensive depth range, feeding in the upper layers at night and excreting in the mesopelagic and upper bathypelagic in the daytime. Like mesopelagic fishes, shrimps thus provide trophic connectivity and transport of organic carbon between the surface and the oceanic deep and could help explain existing discrepancies between flux estimates obtained by the ^234^Th:^238^U method and sediment traps^43^. Indeed, modeling estimates of carbon flux suggest that traps in the mesopelagic underestimate the flux, while deeper bathypelagic traps overestimate flux^44^. Along with fishes, migrating shrimps serve as a bypass, driving a significant portion of organic carbon from surface to deeper layers, whereby particulate organic carbon is not trapped at mesopelagic depths. Instead, shrimp and fish feces increase carbon flux in the upper bathypelagic. As with the case of mesopelagic fishes, underestimated shrimp biomass may also explain unexpectedly large microbial respiration in deep water^45^.

## Conclusions

Tests of two traditional sampling gears, vertical nets and obliquely towed trawls, have shown greater efficiency of vertical nets, which are now recommended for further assessments of shrimp standing stocks. Judging from our results, this methodology will make regressions with environmental factors more robust. We further suggest several considerations for future assessments. Firstly, in cases where a large portion of the water column is sampled (i.e. to 1500 m), shrimps may be sampled irrespective of the time of day; results for the whole water column and for the upper bathypelagic did not statistically differ between night and day. Secondly, the most time-consuming hauls (e.g., below 1500 m) are not critical for the total standing stock assessment, as they usually contribute no more that 4–5% of the total. Thirdly, data of neighboring years may be combined in a single matrix, as interannual fluctuations were not found to be significant on short timescales.
